# Rush Medical College

**Published:** 1867-10

**Authors:** 


### RUSH MEDICAL COLLEGE.

### FORMAL INAUGURATION OF THE NEW BUILDING.

### ALUMNI ASSOCIATION--FIRST MEETING.

### Commencement of the Twenty-Fifth Annual Session.

### A LARGE GATHERING--ADDRESSES BY MAYOR RICE, AND PROFESSORS BLANEY, GUNN, AND ALLEN.

### Supper Given to the Alumni by the Faculty.

ALUMNI ASSOCIATION.

A meeting of the graduates of the college was held October 1st, in the lecture-room of the new building. Dr. J. Blount, of Rockford, was elected Chairman, and Dr. C. B. Reed, of Hampshire, Secretary.

Dr. E. Powell stated the object of the meeting to be the organization of an association of the alumni of the college.

A committee, consisting of Drs. Powell, Ingalls, Johnson, Coleman, and Hunt, was appointed to draw up a constitution and by-laws, and during their absence short speeches were made by Drs. Van Buren, Kennedy, Emmons, and Blount.

A committee, consisting of Drs. Johnson, Kennedy, Blount, McDonald, and Ingalls, was appointed to prepare resolutions relative to the death of the late President of the institution, Dr. Daniel Brainard.

The Committee on Constitution reported, and the report, after alterations, was adopted. It constitutes an association to be called the Alumni Association of Rush Medical College, consisting of graduates in the regular medical course, or those who have received honorary or *ad eundem* degrees, providing they are in good standing in the profession; authorizes at least one literary and social festival each year; fixes the regular business meeting on commencement day, and provides that any member may be expelled by a two-thirds' vote. The Faculty are constituted honorary members of the Association.

The Constitution reads as follows:

*Article 1.* The Association shall be called the "Association of the Alumni of Rush Medical College."

*Art. 2.* It shall consist of graduates, and of those who have received the honorary and *ad eundem* degrees in Rush Medical College, and who are in good standing in the profession, who signify their desire of becoming members of the Association by signing these articles.

*Art. 3.* The members of the Faculty of the College shall be honorary members of the Association.

*Art. 4.* The officers shall consist of a President, 1st and 2d Vice-Presidents, Secretary, Treasurer, and an Executive Committee of three persons, who shall be chosen annually by ballot.

*Art. 5.* The President shall preside at all meetings when present, and perform such other duties as are required by virtue of his office, and at the close of his term of office, shall deliver an address before the Association.

*Art. 6.* In the absence of the President, the Vice-President shall perform the duties of President.

*Art. 7.* The Secretary shall keep a true record of the proceedings of the Association at all meetings, and shall perform such other duties as are required of him by virtue of his office.

*Art. 8.* The Treasurer shall receive, keep, and disburse all moneys belonging to the Association, subject to the order and inspection of the Executive Committee.

*Art. 9.* The Executive Committee shall have charge and direction of the affairs of the Association. They shall take such order as they shall see fit as to any special exercises or proceedings to be adopted for social, commemorative, or festive purposes at any meeting of the Association. It shall be their duty to provide for at least one literary and social festival in each year, when the Association shall meet as members of one family to revive pleasant memories, and exchange new pledges of brotherhood and friendship.

*Art. 10.* These articles may be altered or amended at any meeting of the Association, by a two-thirds' vote of the members present.

*Art. 11.* The regular meetings of the Association shall be held on "commencement day" of Rush Medical College, due notice of which will be given by the Secretary.

*Art. 12.* Any member of this Association, guilty of a violation of the American code of medical ethics, may be expelled by a two-thirds' vote of the members present at a regular meeting; due notice having been given at a previous meeting.

A committee, consisting of Drs. McDonald, Elder, Gueren, Williams, Webster, and Weeks, was appointed to nominate officers for the coming year, and they reported the following nominations, which were unanimously confirmed:—

*President*—Dr. Edwin Powell.

*Vice-Presidents*—Dr. B. F. Spafford and J. Blount.

*Treasurer*—Dr. E. O. F. Roler.

*Secretary*—Dr. W. C. Hunt.

*Executive Committee*—Drs. E. S. Elder, J. F. Weeks, C. Y. Fenn, B. Durham, and T. D. Fitch.

The roll was called, and some seventy of the alumni of the different classes found present.

Interesting speeches were made by Dr. H. Johnson, Prof. Ingalls, and President Blaney, congratulating the institution on its splendid facilities and its prominent future, referring to those of the alumni who had fallen, especially to those who had given their lives while caring for the "boys in blue," and recalling reminiscences of college life, and the meeting adjourned.

THE DEDICATORY EXERCISES.

A large audience of students, medical gentlemen, ladies, and other citizens, was gathered at an early hour in the evening, in the spacious lower lecture-room, the semi-circular seats, rising tier above tier to the height of the second story, being closely filled, making a fine appearance, and showing the general interest felt in the institution.

Many of those present were students just arrived from this and other States to commence the winter course of lectures, and many were alumni of the institution, coming in from their scattered fields of labor to witness and rejoice in the prosperity of their *alma mater*. The platform in front of the amphitheatre was occupied by the Faculty, his Honor, the Mayor, and a number of other prominent citizens.

The exercises were opened with music by Vaas' fine orchestral band, which interspersed the exercises of the evening with their performances.

The President of the College, J. V. Z. Blaney, M.D., then stated that they were assembled upon invitation this evening, assist in the exercises dedicatory of this new edifice, and also to listen to the introductory lecture of the twenty-fifth annual session of Rush Medical College.

Prayer was offered by Rev. Prof. Swing, pastor of the Westminister Presbyterian Church.

PROF. J. V. Z. BLANEY

then delivered the opening address, as follows:—

LADIES AND GENTLEMEN:—This occasion inaugurates what may be termed the fourth epoch in the history of Rush Medical College.

The first epoch was marked by its organization, by the appointment of a Faculty, and the opening of the first course of Lectures, in December, 1843; the second by the dedication of the first building erected for its use, on the site of the present building, in 1845; the third by the enlargement of that building to meet the growing demands of its classes, in 1855; and this, the fourth epoch, is marked by the assemblage this evening of this large and respectable audience to assist in the dedication to the service of medical education of the large and imposing edifice in which you are now convened.

On such an occasion, a brief review of the past history of the institution, and of its early struggles for existence, and of present status among the prominent medical schools of the country will not be considered irrevelant.

The late lamented President of the College—Daniel Brainard, M.D.—on his arrival in Chicago, in 1836, with the view of establishing himself in the practice of medicine and surgery, was early impressed with the opinion that the time would shortly arrive when a medical school would be needed in the Northwest. In the autumn of that year, in connection with the late Dr. J. C. Goodhue, of Rockford, Ill., then resident of Chicago, he drew up the act of incorporation of the College. On the second of March, 1837, this act was approved and became a law. The heavy financial revulsion of that year, which arrested all new enterprises, arrested, with others, the complete organization of the school. Not content with total inanition, as a tentative experiment, Dr. Brainard opened a private school of anatomy in his own rooms on South Clark St., which, with small numbers in attendance, he continued for several years. Meanwhile, he accepted and acceptably filled the chair of Anatomy in the St. Louis University for two years. It was during the session of 1842 and 1843 of that institution that the speaker first met Prof. Brainard, in St. Louis, and learned from him his views in regard to the establishment of a medical school in Chicago; and it was then concerted that should certain contingencies arise during the following summer, a school should be opened in Chicago in the autumn of 1843. Those contingencies were the opening of schools of medicine at several points in Illinois and Indiana. The fact was fully conceded that the movement would be premature, and in advance of the demands of the profession in the Northwest. But it was deemed important, in view of the probability that Chicago, then a town of between 5,000 and 6,000 inhabitants, would continue to be, as she then was, the largest of the numerous towns then struggling for supremacy on the great lakes, that it should be occupied as the site of a medical school, before other schools in other towns should obtain the prestige of priority in their establishment.

A prospectus of a medical school, at Laporte, Ind., and of another at St. Charles, Ill., were issued in the summer of 1843, in view of which it was determined to announce a course of lectures under the charter of 1837, for the winter of 1843 and 1844.

The session opened on the 4th of December, 1843, and the lectures were delivered in two small rooms on South Clark st., to a class of twenty-two members. The Chairs of Anatomy and Surgery were filled by Professor Brainard, that of Theory and Practice by Professor John McLean, that of Obstetrics by Professor M. L. Knapp, and those of Chemistry and of *Materia Medica* by myself.

The second session opened under better auspices, in a building specially erected for the purpose, at a cost of about $3,500, and with a full faculty. Professor Austin Flint, then of Buffalo, N. Y., filled the Chair of Institute and Practice of Medicine, Professor Graham N. Fitch, of Indiana, the Chair of Obstetrics, vacated by the retirement of Dr. Knapp. Professor McLean was transferred to the Chair of *Materia Medica*, and Dr. W. B. Herrick was appointed Lecturer on Anatomy, to which Chair he was elected the following year. The class numbered forty-six, and the graduates eleven. Thus was the Rush Medical College established, and fairly entered on the arena of competition for position as a school of medicine.

Meanwhile, schools had been opened at Jacksonville and St. Charles, Ill., and at Laporte, Ind.; but in the winter of 1847 and 1848, this institution remained master of the field, with a the imperative demands of the overflowing classes which had sought its portals, the faculty determined upon the erection of the noble edifice in which you are this evening assembled—a structure commensurate with the enormous expansion of this great Northwest, and worthy of the important uses it is intended to subserve.

It would not be becoming in me to enlarge upon the weary years of labor expended, the hope deferred, the struggles for life and success experienced in the effort to build up an institution of this kind—prematurely organized, and in a forming and unappreciative community—but I cannot refrain from the remark that much of the position which this College now sustains is due to the foresight which located it in a city, which, by its unprecedented growth, and attainment of universal acknowledgment as the metropolis of a territory unequalled in its resources, present and future, has carried along with it, in its advance, every public enterprise, which, having a worthy object in view, has proved itself adequate to the constantly increasing demands of the communities which are its tributaries.

I should feel that I were unworthy to address you on this occasion, did I not take this opportunity to award the full credit due to the memory of the lamented founder of this institution, for the conception, the inauguration, and the leadership in the successful attainment in the position of prominence, now undisputed, of this college, which was his beloved project from early manhood to the moment of his decease, for whose advancement his whole life of toil was mainly expended, and whose last labor in life was in the duties of the Chair which he had filled from the date of its establishment. There are present in my audience many of the alumni of the College, who still gratefully remember his earnest efforts for their thorough induction into the principles of his favorite department of medical education, and the fervency with which he impressed upon them their duty to assume and sustain a high and honorable position in their chosen profession; and, with me, they will join on this occasion, when celebrating the culmination of his hopes, which, alas, he is not here to enjoy, in awarding all praise and honor the memory of Daniel Brainard. [Applause.]

Another auspicious event which has this day transpired is the organization of an "Association of the Alumni of Rush Medical College." The gratification of those members of the faculty who have enjoyed the high privilege of acting as instructors to the respectable body of professional gentlemen who have this day met to greet their associates of former years, and pay their respects to their *alma mater*, in witnessing the results of their labors, is not to be expressed in words. On more than one occasion, I have had the opportunity to express to classes of graduates the fact that it was upon the success of its alumni, and the position which they should sustain, morally and professionally, in their several communities and among their peers, their *alma mater* would confidently depend for her reputation and success. During the years that have passed, over 1,000 young men have received her honors and passed from her portals to mingle with the class of aspirants for professional success, and make for themselves name and position. The hopes of former years have been fulfilled, and, without fear of contradiction, I dare to assert that, for equal numbers, no institution in the country has greater reason for self-gratulation, whenever and wherever represented by her alumni, than has Rush Medical College. The records of the army of the United States, in its late struggle, the history of every town, village and community in the whole North-west, will furnish evidence which is beyond controversy. This fact, rather than the increasing numbers of our classes, gives to the faculty their highest satisfaction—this their most cherished reward—this their strongest inducement to the sacrifice required in the erection of this noble edifice, destined, as it is confidently hoped, to be filled for years to come with ardent rivals for the attainment of their predecessors.

Confident that they will be sustained by the profession of the whole North-west in their renewed efforts to advance the cause of medical education, the faculty devote all their energies to the work, determined not to remit in sacrifice or exertion until this institution shall take position in the first rank of its peers. [Applause.]

I have now the pleasure of introducing to the audience the Hon. J. B. Rice, Mayor of Chicago. [Great Applause.]

SPEECH OF THE MAYOR.

Hon. J. B. Rice, having been thus introduced, spoke as follows:—

*Mr. President, Members of the Faculty of Rush Medical College, Ladies and Gentlemen:*

A few days since, one of the professors of this college invited me to be present here to-night, to assist in the formal ceremony of opening this important institution. Deeming the invitation to the Mayor, I gladly accepted it, for I desired, as the representative of this city, to show my appreciation of the good work done here by the professors of Rush Medical College, in causing to be erected this substantial, spacious, and commodious building, where medicine and surgery are to be taught.

But my special desire here, to-night, is to join with you all in congratulations over this important event—the building of a college in the city of Chicago that, I am told, and do fully believe, is equal to any similar institution in the United States of America. [Applause.] Then, I would congratulate the city of Chicago that the building has been erected here. I would congratulate the students who are to seek instruction within its walls, that it is erected in a city, the populous metropolis of the North-west, where they will find a people intelligent, frank, kindly, and social, and where the only requisite that is required for their welcome is an honorable character. [Applause.]

A little over twenty years ago, as the President has just told you, the faculty of Rush Medical College delivered lectures to a class of twenty-two students. Last year their lectures were delivered to a class of over three hundred students, and there would have been more to receive the valuable education which is to be gotten here, if there had been room for more. One remarkable part of the history of this college is, and perhaps it is unprecedented, that the entire establishment—all the vast expenditure for its erection—has been borne by the professors of the college. There has been no joint-stock company, and no aid from State, county, or city; no endowments; but the whole sum, seventy thousand dollars, paid by a few earnest men, that the doors of this great building should be thrown open to the thousands of men seeking instruction, from every part of our globe, and coming here where they are sure to find it. [Applause.] This night it is to be dedicated to its use, and I use the term dedicate in its most sacred sense, for, as one of the professors of the college has said, "a broader pile than those of olden time, erected to Æsculapius or Hygiea, shall here arise, for it shall be devoted to truth and humanity." What words! They embrace all the good, or nearly all the good, that we knew in the world. These words were used in promise, but, from the character of the gentleman and his brotber professors, I believe you will all join with me in believing that that promise shall be fulfilled.

Perhaps, medicine and surgery are two of the most important branches of learning in the world. The man who studies and masters them is capable of alleviating pain, of arresting disease, and of prolonging life. The whole human family is deeply interested in the manner in which the men who come here for instruction shall be taught. And here, I hope, the young men who are seeking instruction will not deem me presumptious if I say a word to them. To many of them this place is strange. To all of them it is the place where they are to receive the education which they are seeking. This building is ready prepared for them, and professors, acknowledged to be competent, are ready to impart the knowledge that they seek here. All that is necessary to insure success, is for them diligently, conscientiously, and soberly to pursue the studies that are allotted to them.

I would like to illustrate what I mean, by the recital of a little incident. I once asked a young man, a student at college, a question, for information. Knowing the course of his studies, I thought it was likely he could answer the question. He replied that he could not, that he had once known it, but that he had forgotten it. A gentleman who was standing by (long since dead), a wise man, said that he could not understand how a man could ever forget what he once really knew. That is what I

want to impress upon your minds. I hope that every lesson that is presented to you here, you will receive and grapple with, study and mature, and know, and I believe you will never forget it. [Applause.] If you do this, you will realize the hopes of your parents; you will become the pride of your friends, and the admiration of the public. And every one now present hopes you will do so. They feel a deep interest in this matter, as is evinced by their presence here to-night.

I sincerely hope and believe that everything that is done within the walls of this college will be wisely and well done, for the benefit of the people, for the benefit of its pupils, and for the honor of its faculty. [Much applause.]

PROFESSOR GUNN.

Moses Gunn, M.D., Professor of Principles and Practice and of Surgery and Clinical Surgery in the college, then addressed the assemblage as follows:

GENTLEMEN:—

In behalf of my colleagues, I bid you welcome! Welcome to Chicago; the young giant of the West! Welcome to Rush Medical College, and to these halls which we this day dedicate to Science and Humanity.

You compose the twenty-fifth class which has annually assembled here, on what has become classic ground, seeking after truth in medicine; truth ever simple and yet often elusive; which lies not unfrequently immediately before us, while with strained vision we attempt to pierce the dim and smoky distance to discern it; which from its very simplicity is oftentimes completely hidden from a search which looks for it only enshrined in deep and difficult mystery.

Annually, for the past twenty-five years, in search of this gem have your predecessors come up hither; with what of success let their individual history in the teeming North-west, with its cities, villages, and expanded plains, and its ever increasing population, and, also during the late protracted and bloody war, tell.

Twenty-five years! In the longest life an extended measure; in the history of human events a pitiful period; and yet, in the early history of a city or a nation, how important! And if measured by what is sometimes accomplished, how the little quarter of a century seems to sink its fractional character and assume the dignity of the full and unbroken unit.

Measured by her growth and achievements, Chicago might well rise to the full period of a century! What was she when the first little class assembled here under the auspices of the then infant College?

An old military post on the extreme North-western frontier had but recently became recognized as a town. Westward stretched the rich and undulating plain, on to the father of waters. Eastward the great chain of lakes afforded communication with the older cities of the continent. Aside from this summer avenue, the plodding team of the emigrant, and the mail of our venerable and common Uncle Samuel, transported as the exigencies of the season and condition of the roads would permit, constituted the only means of communication. Nestling on either side of the bayou lay the infant city. No broad avenues stretched off for miles over the plain, but low upon the oozy surface of the original prairie lay the yet limited streets. Reared upon posts stood the young city, the whole aspect verifying the need which found expression at a much later date, when the characteristic enterprise of the inhabitants rendered the idea not wholly improbable, to-wit: The issuing of proposals and inviting bids for a young earthquake to elevate the site to a desirable altitude. No railway network stretched out to the boundless regions on all sides, bringing to a common centre the more than Indian wealth of the country, and making here a granary for half the world. No temples of trade crowded compactly the streets, and upward reached for more ample accommodation.

No tunnel, at once the wonder and triumph of art, penetrated for miles under the majestic lake to draw from its chrystal fountain health and happiness for half a million.

No medical halls such as we this day dedicate invited such a class as we now welcome; and no Æsculapian orator had caught the spirit of place-glorification, which outside barbarians assert to be the sign diagnostic of a Chicagonian, and held forth to the first class here assembled with that peculiar and diagnostic modesty. But, *In hoc signo vinces.*

On the contrary, a small and unpretending building occupied the spot; a little class of twenty-two students assembled here, and while the primary faculty were honestly, and earnestly, and successfully initiating this great enterprise, they dared not dream of the magnificent future. To that first faculty I would here acknowledge in behalf of the whole profession our great indebtedness. But one of that little band remains with us, and he, honored among all, is our crown of rejoicing.

But if on this ground has grown from a small beginning a great city and a great medical college, so that as compared with the original, the present appears to be a full fruition, we shall find that change and improvement are not confined merely to city and college. The science and art of medicine and surgery has, during the period which we are contemplating, made such advances as to elicit the admiration of him who watches its history, and to excite the pride of its votaries.

The student who sat under the first course of lectures in this institution, could he be transported over the interval without having participated in the advance of the profession, would find himself utterly bewildered and unable to understand much that he would hear in the course of instruction as now given. While the whole scientific world has been pressing forward in pursuit of undiscovered truth, medical men have not been surpassed in industry and zeal, nor have the fruits of their labors been few or scanty.

In chemistry alone a new science has almost been created. Old fields have been reworked and new ones explored; and not content with the elements and organisms of earth as presented in its vast laboratory, swallowing up bodily the new science of geology, and illustrating that its evidences are but the result of chemical reactions in old earth's chronology, the chemist has pushed his investigations into other spheres, and in his spectral analysis vies with the astronomer in the study of those remote fields. The domain and laboratory of chemistry is the universe!

Within this period the microscope has mainly wrought out its great work, and Histology now claims its own peculiar dignity. Under its ministrations, too, Physiology and Pathology have extended their bounds and refined their processes.

Physiology then was dispatched in a few crude lectures, and these were usually given by the anatomist. The physiology of nervous action had then to offer as its latest and brighest work the reflex-motor action of Marshall Hall, which the intelligent physiologist of the present day knows to be but a single phenomenon in the list of reflex actions. The reflex influence of impressions upon organic changes, nay, the reflex influence of those changes upon other functions of nutrition; and the reflex influence of the normal processes of local nutrition upon one another;. the influence of mind upon matter and matter upon mind are but the operation of the same law. An elaborate paper announcing the discovery of the reflex secretory action of the nervous action was presented to the American Medical Association, at its session in 1857, by Prof. Campbell, of Georgia. Marshall Hall, himself, admitted the discovery, and hailed it as a twin companion to his own, thus publicly complimenting his young American brother.

But it is within the knowledge of your speaker that the whole subject of reflex nervous influences, of which excito-motory and excito-secretory actions are but constituent parts, was taught as early as 1850 in the University of Michigan by the present incumbent of the chair of practice of medicine in this institution, Prof. Allen. In his teachings and writings, too, are to be found the only explicit and comprehensive exposition of the whole subject of reflex nervous action that has ever fallen under my observation.

Fresh then, were the experiments of Beaumont upon the stomach of the soldier, Alexis St. Martin, which interesting and valuable as they were, have required the scrutiny of subsequent analysis to correct many of the first conclusions and expunge not a few gross errors.

Therapeutics as a *science* has almost been born within this period. While Materia Medica was as colossal (God save the mark!) then as now, the philosophy of the action of remedies, not mere medicines, has claimed paramount attention, and general Therapeutics to-day commands far more study than mere materia medica.

Pharmacy, the hand-maid of materia medica as taught and practiced to-day, would hardly be recognized by a member of the profession who had indulged in a Rip Van Winkle nap. Crude processes and gross preparations have been supplanted by delicate manipulations and the active principles of medicines. The doctor no longer bestrides his Rosinante with his pannier-like saddle-bags stuffed with the crude *materiel*, nor does the table in the sick-chamber look like an apothecary's counter. Organic chemistry enables the pharmaceutist to fill our prescriptions with efficient, concentrated and non-repulsive remedies.

Practical Medicine has, also, during the period which we are considering, undergone a no less marked change and improvement. A more general and at the same time clear, definite, and intelligent view, and application of nervous influence upon normal and abnormal action; and the use of such influence in allaying disease and promoting health; a more confident reliance on inherent recuperative power, and the ability to excite, control, and modify that power and marshal its force, and command its aid in the cure of disease; a much more guarded resort to powerful and uncontrollable means and depleting medicines; a growing tendency to look to the general condition of nutrition as a means of cure, as, for example, in the management of phthisis and kindred conditions of the system; the influence of pure air, cleanliness, and light, as seen in the management of hospital wards, and sick chambers, in private dwellings; all these considerations mark the progress in medicine since the days when Eberle wrote, and the mass of the profession in this country followed his directions, or, if differing with him, still relying as confidently as he on the mysterious power of medicine to combat and cure disease.

Surgery, too, has felt the influence of the times. First, in importance, as well as chronologically, is the discovery of a means of producing a state of complete anæsthesia, a discovery which was the dawning of a new era in surgery. Not merely the ability to perform operations without pain to the patient, or even to perform at will, hitherto almost impossible operations, constitutes the limits of this discovery. The relaxation which attends its full state, is a condition of the system which is oftentimes most desirable, and which was formerly sought to be established, in many instances, by a resort to nauseants, venesection, and the hot bath. Unconsciousness is at the same time frequently desirable, and in this double effect, are the power and influence of anæsthetic agents at once grateful to the patient and valuable to the surgeon. The honor of this discovery is American. Whether to Drs. Wells, Morton, or Jackson, individually, appertains the immediate credit, it is not my purpose to inquire, it is sufficient that it belongs to the period of time which marks our history, and that it is American.

That department of surgery which appertains to the eye, has also been marked by the most note-worthy advances. The ophthalmoscope, alone, has wrought great changes. It has opened up as rich placers as did the stethescope of Laænnec in another department, and at an earlier date.

Still more recently, the laryngoscope has enlarged our means of observation in another field; while the endoscope, with still greater enterprise, enables us, almost,

"With optics sharp, I ween,
To see what is not to be seen."

The late war has also afforded means for successful study, which have not been neglected, and the accurate observations in reference to the pathology of pyæmia and hospital gangrene, have resulted in such a development of their pathology, as to direct to a rational and eminently successful treatment, both prophylactic and curative.

In all departments of our profession, progress has been the watchword, and in those branches which more nearly approximate the fixed sciences, and which, consequently, afford fewer opportunities for advancement, improvement in method, and refinement in process have been as marked and decided, as discovery has been in others.

But, Gentlemen, I have not thus alluded to the achievements of our science in a spirit of vainglory. I would not underrate the labor and advances of any period previous to the last quarter of a century. I would not even attempt a comparison which might be deemed invidious between any period and that which I have contemplated. Each century, and even each decade, has had its own success and glory, and stretched along the whole history of medicine, are the records of labor, some plainly and unmistakably saying to us, This is the way, walk ye in it; others, like beacon-lights, warning us off the rocks and sands of error. And so it must continue to be. As long as there is yet a truth to be discovered, many failures to a single success must occur. But for every success there is ample reward, though accompanied by a thousand failures.

I have indulged in the line of thought which I have followed, rather to encourage and stimulate hopeful effort on your part. By so much as we have advantage over those who have preceeded us, may, and probably will our successors realize improvements upon us.

Appreciation of ancient truth does not demand of us unbounded credulity. It does not require us to accept as truth all that is ancient, simply because it is venerable; neither does it expect us to shut our eyes upon the glory of the present because it has not the dust of ages upon it. There is a class of men, represented largely in our profession, whose veneration is profound, and leads them to see no good in the present, except that it was born in the past; who constantly exclaim there is nothing new under the sun. From such veneration, in the language of the litany, "Good Lord deliver us."

But, on the other hand, appreciation of modern discovery does not require us to sneer at the past because its measure was not full; nor should we make the mistake of regarding our own period as the culminating point in the history of medicine. That point, gentlemen, will never be reached. The grand day of science will have no declining sun; but the glorious orb of truth will ever rise higher and higher, and shine with ever increasing refulgence, until the universe shall be lighted.

When shall that be? When *all* shall be known; when we shall know even as we are known. Would you estimate the period by the lapse of ages? Attempt if you can to conceive of the amount yet unknown, and when your mind can begin to take in that conception, then commence to measure the day of science.

We are yet but in its early morning, a morning to be succeeded by no noon, no evening, but by an ever brightening day. With this conception of the situation, with this idea of the relation of the present to the future, there is no ground for indulgence in vain glory; for vain indeed would be any glorification which forgets for a moment the littleness of the present, when compared to the probabilities of the future. As our perceptions of temperature are relative only, so our estimate of the present state of science must be relative. It may be great compared with the past, but what is it when we look forward to the possibilities of the future? It is yet the day of small things, and our pleasure as well as our duty, should be to work earnestly, as opportunity offers, and opportunity is not rare; we can hardly miss it: our fault is rather in a disposition to select from the mass, than avail ourselves of that which is immediately before us. Whatsoever thy hand findeth to do, do it with thy might. Work is and must be the motto and lot of every successful man; it is so, *par excellence*, with the student in the science of medicine. There is no short high road to advanced learning; but by study, thought, experiment, and observation, the race must be won.

Most medical men study more or less; they also are, as a general rule, good observers; a few experiment, but, alas, how very few seem to *think!* and I confess that it has sometimes occurred to me that those who conduct large series of experiments seem to think the least. I have spoken of medical men because I am speaking to you who are to become such; but I would not be understood as criticising my professional brethren, as peculiarly disinclined to reflect. The remark applies to all men. Mankind is prone to accept the *seeming* rather than search out the real; to accept a received explanation rather than laboriously to critisice it. It is not enough to study, to observe, to experiment, we must do more; and in this connection let me impart this injunction: *think!* whatever you do, *think;* study, but *think;* observe, but *think;* and especially, if you experiment, *think*.

It is easy, by study, to possess yourself of the thoughts of others, to appropriate, assimilate and make them your own; but you may do this without ever indulging in the luxury of a thought of your own. You may observe extensively, and yet, like a crab, you shall even go backwards for not pondering well upon what you have observed. You may experiment till you draw down upon your devoted heads the persecution of a sentimental Bergh and his colaborers, who are themselves an example of observation without thought, and yet never penetrate deeper than the simple fact or phenomenon which is the immediate result of your experiment.

Therefore, gentlemen, I again repeat, *think*. Think for yourselves; contract the habit of thinking, and with the practise will come increased ability to study, observe, and if you choose, experiment. But while thought will not take the place of study and observation, it is the soul of both. Without it, either study or observation is the Adam into whom the breath of the living spirit has not yet been infused. It is the ovum without fecundation, destined only to blight and decay.

But, perhaps, I should be more explicit in this injunction. Men differ in intellectual power, and, in accordance with this general proposition, you are not all mentally equal. To one is awarded only mediocre powers, while on another are bestowed both brilliancy and profundity. Neither are you all equally advanced in education. To some, the advantage of free and generous culture has been abundantly given, while others are struggling in their course with the impediments incident to an imperfect education.

In medical advancement, too, you will be found to be widely different. Some are but just entering upon their course, while others are well advanced, and are more or less familiar with all matters appertaining to medical and surgical science. It is evident that the ability to think correctly and advantageously upon the various subjects of your study and observation will vary with the different orders of intellect, and the varying degrees of culture. Still my advice applies to all. None need be so deficient, if at all qualified to pursue medical studies, as to accept all that he hears, reads, or observes, as truth, so positive and unqualified as to need no other effort of the mind than that involved in the act of appropriation. The merest neophyte though incapable of calling in question anything that is presented to his understanding, should, by earnest thought, endeavor to detect the reason for whatever he hears, reads, or observes. Not only will this mental process fix the subject of this thought, and constitute in itself the most perfect means of assimilation, but it will prove a method of mental training that will develop power and facilitate future effort.

As the student advances in his course, and attains a standard of acquirement that gives him a stock of well established and undoubted truths, he should, in addition to the search for the reason of things, compare his result with these standard truths, and thus another step in advance is taken. His stock of the actual is constantly increasing; and not the actual only, but the reason thereof, and the relation which it sustains to other facts and phenomena. Prove all things, hold fast that which is good. Accept nothing simply because you read it, or hear it, or, even, *see* it. Subject all things to careful mental analysis, and, finally, believe, not because you have read, heard, or seen, but because it is recommended, *per se*, to your individual judgment. Let your religious belief be a matter of faith, but let me warn you against receiving your scientific creed on the same basis.

Faith is an excellent quality in your patient, for oftentimes he will be obliged to indulge in the substance of things hoped for, the evidence of things *not* seen; but on your part, it will be better both for science and humanity that you believe nothing, literally nothing, till proven.

As students, sitting at the feet of your Gamaliel, even though you may have good reason to distrust your own judgment, and feel greater, far greater confidence in the author you read, or the professor you hear, still make the effort to go through with the process which I have recommended, before finally deferring and believing.

You will thus strengthen your own power and gradually acquire independence and accuracy of thought. You will acquire, too, the power of discrimination; a power of weighing and comparing before deciding. The medical man finds a great amount of conflicting, or apparently conflicting evidence, and like the jurist, he must weigh, compare, sift out, reject this, and accept that, all, too, in accordance with established law. Thus the law becomes purified by rejection and amplified and perfected by slow crystalization. Our circle of knowledge is expanded and the domain of truth is enlarged.

Another habit of thought should also be cultivated, *viz.:* the seeking after the *soul* of the truth. As there is a soul of goodness even in things evil, there is an innermost kernel to all subjects, an element by which and through which it differs from all other similar subjects with which it might be confounded; it is in virtue of this element that the truthfulness consists. A clear and definite conception of this element only, will enable you to master a given subject, and so long as you fail to detect it, the real truth remains hidden from your view. A loose, general, and vague idea you may have, even as one sees an object, through a foggy atmosphere, without being able to discern its exact form, its individuality. It is the fault of many minds to be satisfied with such a view, and to neglect the labor incident to the full defining of the picture. It may be that all effort will fail in some instances to bring out the details clearly; but the effort should, nevertheless, he made to attain a clear and definite perception of what I have termed the soul of the truth.

To illustrate: Volumes have been written, and more said on the subject of inflammation; all the phenomena thereof have been enumerated and the changes rung on them. Concise definitions have been attempted and criticized; and by some it has been said that such a thing as a correct definition of the subject was an impossibility. All observers, of any experience whatever, recognize an inflammation when they see it, and yet many will fail to discern in just what it consists.

Let us now make an effort to obtain a view of some one circumstance in reference to inflammation, by which it differs from all other similar conditions. It is not pain, heat, redness, nor swelling, nor all of them that constitutes the condition under consideration, for any one or even all of them may be present without the part being in such a state.

The blush which mantles the cheek of shocked and offended modesty, when extreme, may cause it to burn and tingle with heat and pain, while the actual engorgement of the vessels supplies the redness and swelling.

Blood may flow in greatly increased quantities *to* and *through* a part, invited to do so by an increased activity of normal local nutrition, producing even active hyperæmia. This may serve a temporary and useful purpose, examples of which will occur to the mind of any medical man; or, it may, if long continued, result in hypertrophy of a part; but so long as the local nutrition is only stimulated and increased in activity, so long as the advance and retrograde changes exactly balance each other, there is no inflammation. But the instant that this active hyperæmia is attended by oppression and impairment of local nutrition, inflammation begins, and in this impairment or suspension it consists; and just so soon as normal local nutrition is again established, inflammation has ceased, even though active hyperæmia may yet remain. This is the key to the whole subject. *It is the soul of the truth.*

Another illustration: You attempt the study of *ulceration;* you read author after author; you watch the process at the clinic, and the probabilities are that you will obtain a confused idea of disintegration of tissue, mortification in miniature and absorption, attended by suppuration. Confusion worse confounded! But careful observation of the process, in numerous instances, and correct analysis of what you observe and read will clear up the subject, and isolate the identical characteristic of ulceration. Disintegration of tissue in particles, or mortification in miniature is not ulceration, for, pathologically, mortification is the same whether in miniature or on a colossal scale. Suppuration, although a frequent attendant on ulceration, is not a necessary part of the process; but by observing the ulcerative process you will see that tissue disappears, sometimes without crumbling away by mortification in miniature or even being attended by suppuration. What has become of it? It is not volatile, and can not have vaporized; only one other method of disappearance is left, and that is by absorption; an absorption that destroys the integrity of tissue; and here we arrive at the isolated characteristic of ulceration, *viz.*, *destructive absorption of tissue.* In this it consists and in no other process.

Thus, gentlemen, you should *think; reflect* upon each and every subject which you enter upon, and endeavor to arrive at the *soul of the truth.*

But there is one matter especially which I earnestly recommend you to carefully consider and endeavor fully and perfectly to comprehend. It is expressed in the answer to the question, what is disease, and how can it be prevented, alleviated, cured?

I do not purpose to attempt an answer to this question at this time. That answer will be found permeating the whole course of instruction which you will receive in this College.

But I warn you against regarding disease as a subtle essence which invades and permeates the animal being, to be charmed away by incantations or other spiritual means, on the one hand; or on the other, as a hydra-headed monster which, in various forms, enters the fair citadel, to be ejected only by medicinal potations, either great or small. Learn rather to look upon the human fabric as a delicate organism which for a time is the seat of a vital force, which, springing from the throne of the Omnipotent, wrests matter from the action and sway of mere chemical affinities, seizes chemical laws and harnesses them to its own work; permitting them to have full and unrestricted action in one place, modifying and controlling them in another, while in still another .they are bound hand and foot; and the elements, like the lion and the lamb, are made to lie down together in peace and harmony.

This intricate and delicate organization is the seat of numerous functions all subservient to the existence of the whole, and playing upon one another by a system of direct and reflex influences, which, in harmonious action, conserve the object of its creation — a *limited* existence.

A derangement in any single function exerts its influence upon others, and thus, by destroying the harmonious action of all, tends to shorten even the natural limit of existence. *This is disease.*

To learn to detect it in its primary, and all its secondary lesions, and to correct it — to recognize the causes which produce it, in order to obviate them, is your mission. In that mission, I bid you *God speed*, and in behalf of my colleagues, I pledge you our hearty coöperation and assistance.

### THE SUPPER.

Professor J. V. Z. Blaney then announced that the regular lectures of the coming course would begin at 9 o'clock in the morning, and stated that the faculty had prepared an entertainment for the *alumni*, in a room above, to which they would invite the trustees, members of the regular profession, the College of Pharmacy, and the students.

The benediction was then pronounced by the Rev. Professor Swing, and those included in the invitation repaired to the dissecting-room, where the deceased bodies of fowls, pyramids of ice cream, cake, and other delicacies, were spread out for dissection.

Professor Allen took the head of the table and did the honors in a short speech.

After a time spent in social conversation, the company dispersed, mentally and physically gratified with the exercises of the evening.

### A HANDSOME GIFT.

A fine set of surgical instruments, worth $700, has been presented to the institution by Tieman, the celebrated manufacturer, of New York, and was on exhibition in one of the rooms, to the admiration of the profession.

